# Supplementary figures and images for: Efficacy of Supplementation with B Vitamins for Stroke Prevention: A Network Meta-Analysis of Randomized Controlled Trials
Source: PLoS One. 2015 Sep 10;10(9):e0137533. doi: 10.1371/journal.pone.0137533 (PMC4565665; doi:10.1371/journal.pone.0137533)

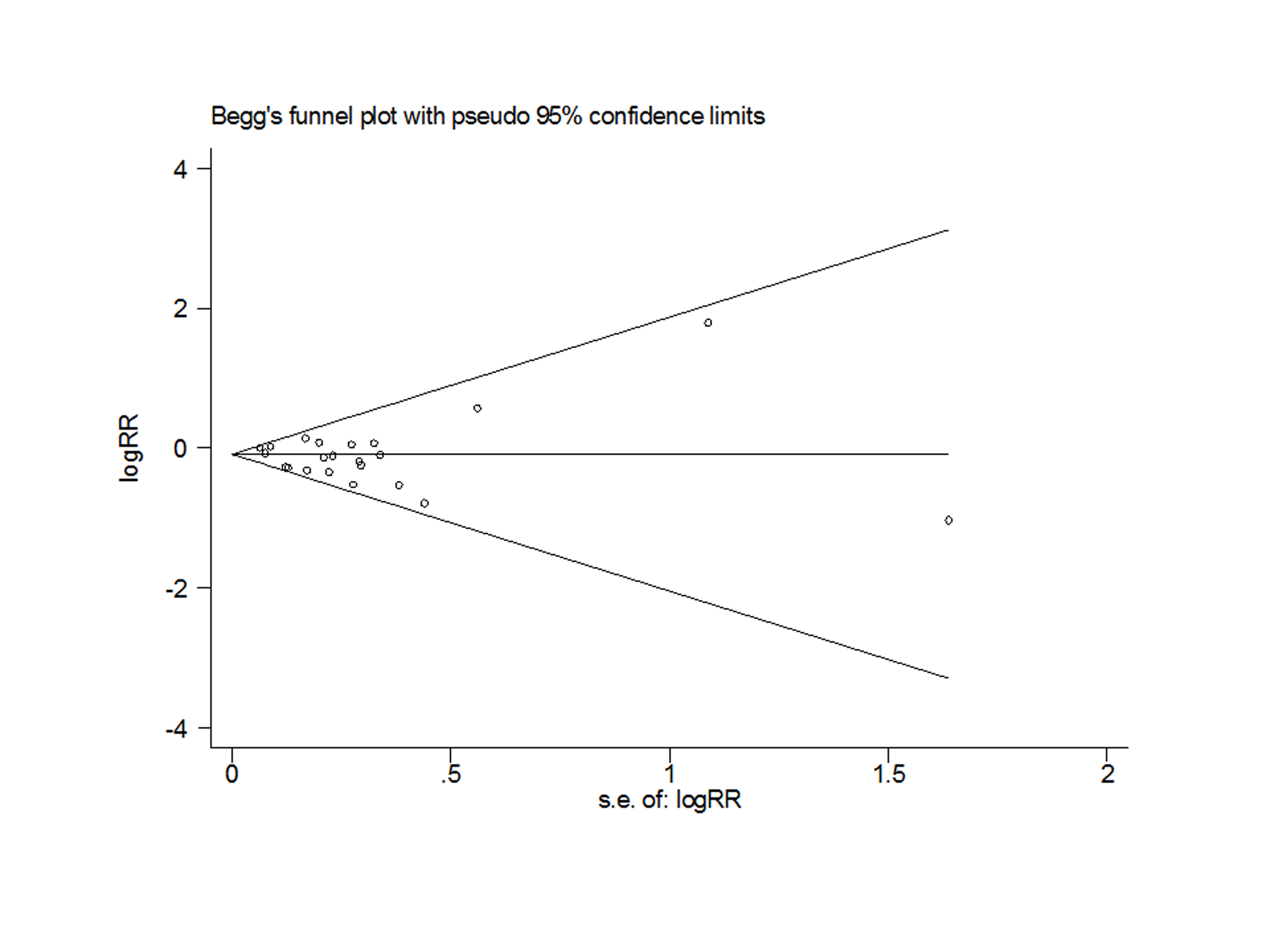

Supplement: S1 Fig — (TIF) [file pone.0137533.s001.tif]

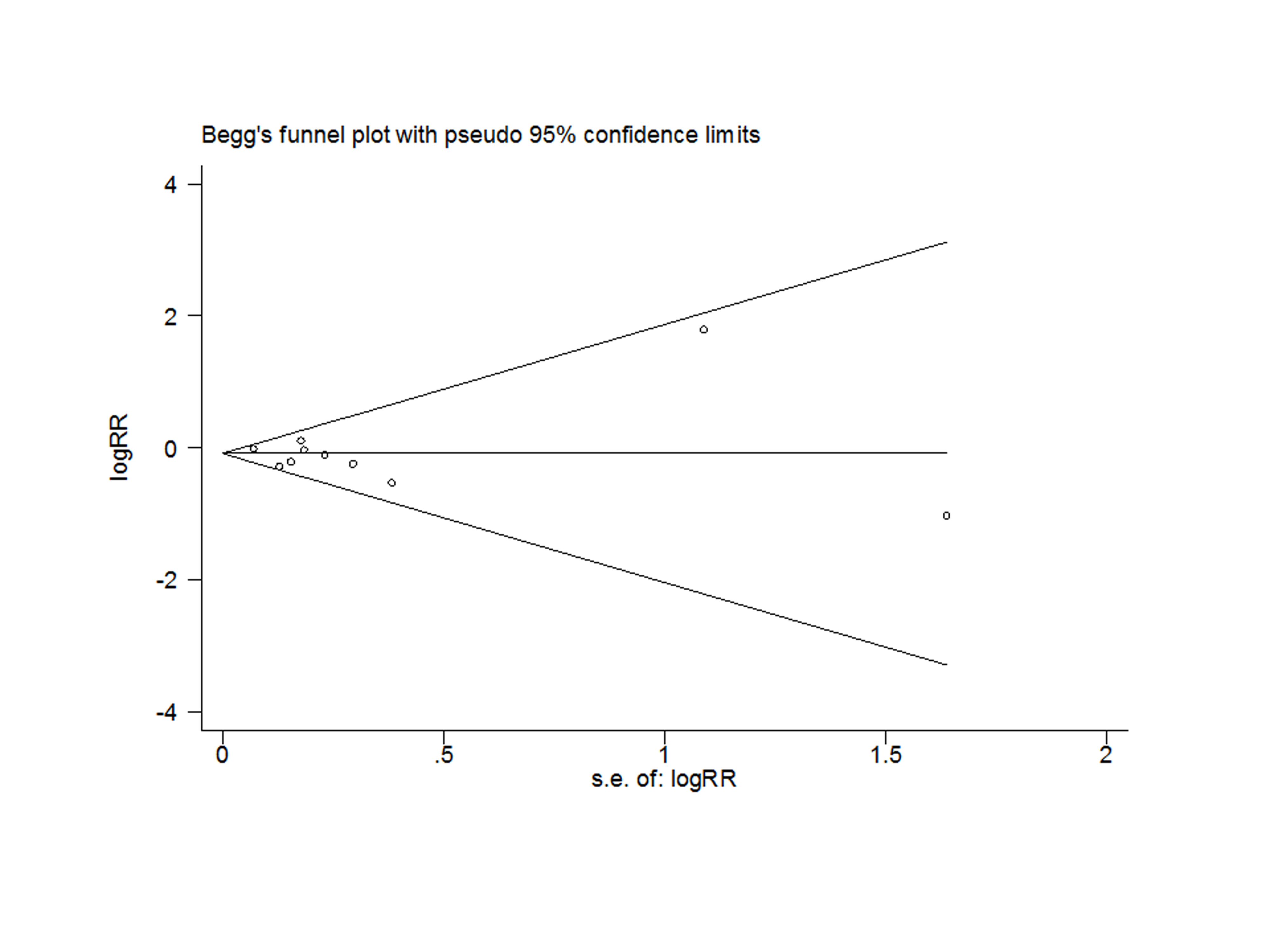

Supplement: S2 Fig — (TIF) [file pone.0137533.s002.tif]

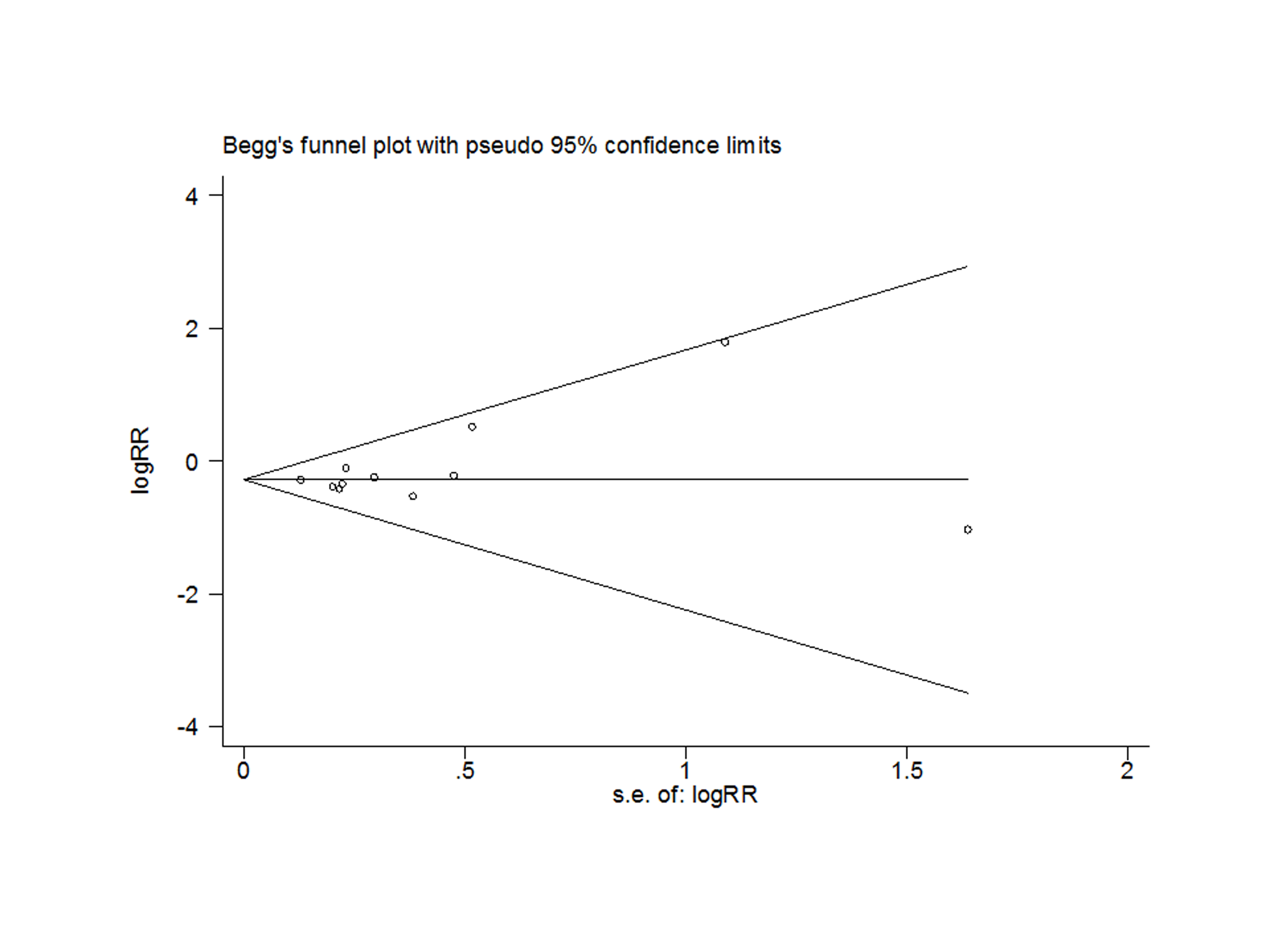

Supplement: S3 Fig — (TIF) [file pone.0137533.s003.tif]
