# Supplementary material for: Efficacy of Supplementation with B Vitamins for Stroke Prevention: A Network Meta-Analysis of Randomized Controlled Trials
Source: PLoS One. 2015 Sep 10;10(9):e0137533. doi: 10.1371/journal.pone.0137533 (PMC4565665; doi:10.1371/journal.pone.0137533)
Supplement: S1 PRISMA Checklist — (DOC) [file pone.0137533.s004.doc]

| **Section/topic** | **#** | **Checklist item** | **Reported on page #** |
| --- | --- | --- | --- |
| **TITLE** | | |  |
| Title | 1 | **Efficacy of** **supplementation with B vitamins for stroke** **prevention:** **A network meta-analysis of randomized controlled trials** | #1 |
| **ABSTRACT** | | |  |
| Structured summary | 2 | **Background** Supplementation with B vitamins for stroke prevention has been evaluated over the years, but which combination of B vitamins is optimal for stroke prevention is unclear. We performed a network meta-analysis to assess the impact of different combinations of B vitamins on risk of stroke.  **Methods** A total of 17 trials (86 393 patients) comparing 7 treatment strategies and placebo were included. A network meta-analysis combined all available direct and indirect treatment comparisons to evaluate the efficacy of B vitamin supplementation for all interventions.  **Results** B vitamin supplementation was associated with reduced risk of stroke and cerebral hemorrhage. The risk of stroke was lower with folic acid plus vitamin B6 as compared with folic acid plus vitamin B12 and was lower with folic acid plus vitamin B6 plus vitamin B12 as compared with placebo or folic acid plus vitamin B12. The treatments ranked in order of efficacy for stroke, from lower to higher, were folic acid plus vitamin B6 > folic acid > folic acid plus vitamin B6 plus vitamin B12 > vitamin B6 plus vitamin B12 > niacin > vitamin B6 > placebo > folic acid plus vitamin B12.  **Limitations** Firstly, we may have missed some data from unpublished studies, which might have affected our results. Secondly, we considered only the outcomes of interest in all eligible studies rather than the specific information for participants in different intervention groups in our network meta-analysis.  **Conclusions** B vitamin supplementation was associated with reduced risk of stroke; different B vitamins and their combined treatments had different efficacy on stroke prevention. Folic acid plus vitamin B6 might be the optimal therapy for stroke prevention. Folic acid and vitamin B6 were both valuable for stroke prevention. The efficacy of vitamin B12 remains to be studied.  No systematic review registration number. | #2 |
| **INTRODUCTION** | | |  |
| Rationale | 3 | Stroke is the second leading cause of death in the world and the common cause of disability-adjusted life years lost globally [1,2]. Observational studies have demonstrated high total plasma homocysteine levels in patients with stroke [3], and elevated homocysteine levels could increase the risk of stroke [3-6]. Supplementation with B vitamins is used to lower the increased plasma homocysteine level to prevent cerebrovascular disease [7]. Although many studies have implied an association of B vitamin supplementation and risk of stroke, the effects of B vitamin supplementation on stroke prevention are not consistent. Comparisons of the effect of different combination of therapeutic methods, such as folic acid plus vitamin B6, folic acid plus vitamin B6 plus vitamin B12, vitamin B6 plus vitamin B12, folic acid plus vitamin B12, are insufficient.  Network meta-analysis can simultaneously combine both direct and indirect evidence from studies and compare all existing therapeutic methods to assess the relative efficacy of each treatment on the basis of randomization [8-10]. It can better assess the relative effects of different B vitamins and their combinations on stroke prevention than the standard head-to-head meta-analysis. | #3 |
| Objectives | 4 | In this study, we performed a network meta-analysis of randomized controlled trials of B vitamin supplementation to identify the optimal therapy for preventing stroke, including cerebral infarction and hemorrhage, and compare the relative efficacy of supplementation with different B vitamins and their combinations. In addition, we ranked B vitamins and their combinations to help patients and clinicians choose reasonable and effective B vitamin interventions for stroke prevention. | #3 |
| **METHODS** | | |  |
| Protocol and registration | 5 | No protocol and registration. |  |
| Eligibility criteria | 6 | We included studies that (1) were randomized controlled trials evaluating the effect of different B vitamins and their combination on stroke; (2) compared B vitamins and their combination with a placebo; and (3) had a length of follow-up of at least 6 months and outcomes of study included stroke. We excluded (1) gene polymorphism studies, case reports or case series and animal studies and (2) trials that did not report the outcomes of interest or clarify stroke events. | #4 |
| Information sources | 7 | We performed a literature search of MEDLINE via PubMed for studies published in any language through November 2014. | #4 |
| Search | 8 | The following combination of MeSH terms and free text words: “homocysteine” or “folate” or “folic acid” or “pteroylglutamic acid” or “vitamin B6” or “pyridoxine” or “vitamin B12” or “cobalamine” or “cyanocobalamin” or “niacinamide” or “3–pyridinecarboxamide” or “B vitamins” or “neurobion” and “stroke” or “brain infarction” or “cerebral hemorrhage” or “subarachnoid hemorrhage” or “intracerebral hemorrhage” or “cerebrovascular disease” or “cerebrovascular attack”. We restricted our search to randomized controlled trials and humans. In addition, we reviewed reference lists of published meta-analysis or reviews to identify additional eligible studies. The last search was on November 2014. | #4 |
| Study selection | 9 | The titles and abstracts of eligible articles were reviewed independently by two investigators (HLD and DZ) with a standard protocol, then the full text was read. Any discrepancies were resolved by discussion or consultation with a third investigator (SJP). | #4 |
| Data collection process | 10 | The following data were extracted by two investigators (HLD and DZ): first author or study group’s name, country, publication year, study design, type of blinding, length of follow-up, patient characteristics, number of stroke events and interventions. | #4 |
| Data items | 11 | Relative risk (RR) and 95% confidence intervals (95% CIs) were computed. | #5 |
| Risk of bias in individual studies | 12 | Despite a detailed search mechanism, we may have missed some data from unpublished studies, which might have affected our results. As well, we considered only the outcomes of interest in all eligible studies rather than the specific information for participants in different intervention groups in our network meta-analysis. | #12 |
| Summary measures | 13 | We performed the standard meta-analysis by adopting fixed- and random-effects models to compare B vitamin supplementation versus placebo for the outcomes stroke, cerebral infarction and cerebral hemorrhage. Relative risk (RR) and 95% confidence intervals (95% CIs) were computed. According to Begg test, the publication bias of trials was assessed in the regular meta-analysis (*P* > 0.05 was considered as no publication bias). Sensitivity analysis was conducted to test the reliability of the pooled results. Heterogeneity which quantifies the proportion of total variation attributable to genuine between-study variance rather than random error, was assessed by the inconsistency statistic (*I^2^*) and *P* value for chi-square statistics; *I^2^* < 25% or *P* > 0.10 was considered as low heterogeneity and *I^2^* > 50% or *P* ≤ 0.10 as high heterogeneity [12].  For the main analysis, we performed a Bayesian network meta-analysis, combining all available direct and indirect treatment comparisons to evaluate the effect of B vitamin supplementation between all interventions and ranked different interventions [13]. Based on Bayes theorem, Bayesian network meta-analysis was generally available using the Markov chain Monte Carlo software WinBUGS, which sorted all intervention measures and got accurate results based on the posterior probability of the Bayesian method. Network plot was established in the network meta-analysis, which offered a visual representation of the direct and indirect treatment comparisons and a concise description. For the network meta-analysis, outcome variables were compared by a random-effects model because its variance includes variation between groups and within groups, which generally showed more conservative estimates and better goodness of fit than a fixed-effect model. We used the Markow Chain Monte Carlo method (the number of initial iterations to burn in was 10 000 and the next iterations for estimations was 30 000) to estimate each strategy. The goodness of fit of the model was evaluated by deviance information criteria and assessing the posterior mean of the residual deviance. When residual deviance approaches the number of data points, the model has good fit [14]. In general, the posterior distribution of the parameters of the outcome of interest was shown with RRs and 95% credible intervals (95% CrIs). In addition, the posterior probability of the Bayesian method was used to calculate the RR for each regimen compared with a placebo to evaluate the probability that one B vitamin intervention was best in terms of stroke prevention, second best, third best, etc. The difference between direct and indirect evidence of all closed loops was calculated in the network; inconsistent loops were assessed by inconsistency factors (IFs) compatible with zero or 95% CIs including 0, which indicated no significant disagreement between direct and indirect evidence [15]. The percentage contribution of each comparison group on the network meta-analysis was assessed in a matrix with rows and columns. The rows refer to the possible pairwise comparisons from the process of analysis and the columns refer to the direct comparisons from original data. Based on direct comparisons to mixed and indirect estimates, respectively, the differences between direct comparisons which affect their relative contribution to the network meta-analysis were assessed. The overall estimates were indicated by entry of entire network, which reflected overall contribution of each direct comparison group to the network estimates. | #5-#7 |
| Synthesis of results | 14 | **Eligible trials**  17 articles (14 two-arm trials and 3 four-arm trials) were included in the network meta-analysis; 86 393 patients were randomly assigned to 8 different intervention groups: FA + VB6, FA, FA + VB6 + VB12, VB6 + VB12, niacin, VB6, placebo and FA + VB12 (**Fig. 1**). The follow-up ranged from 0.5 to 10 years and the age of patients from 18 to 80 years. The main characteristics of the studies are in **S1 Table** [16-32]. The overall study quality assessed by the Jadad score was considered high (**S2 Table**).  **Traditional meta-analysis**  As compared with placebo, supplementation with B vitamins was associated with reduced risk of stroke and cerebral hemorrhage in the standard meta-analysis (**S3 Table**). We found no association of B vitamin supplementation and placebo for patients with cerebral infarction. Meanwhile, we found no significant heterogeneity for studies of stroke, cerebral infarction and cerebral hemorrhage (all *I^2^* < 50%, *P* > 0.1) (**S3 Table**). According to Begg test, the publication bias of trials was assessed in the regular meta-analysis, including stroke group, cerebral infarction group and cerebral hemorrhage group, respectively. There was no obvious bias in each group (*P* > 0.05 was considered as no publication bias). Begg's funnel plots were shown in the Supporting Information (**S1-S3 Figs**). In addition, we performed sensitivity analysis of 22 trials for stroke prevention, in which one study was removed at a time, to evaluate reliability of the result. When the studies by Saposnik et al. and House et al. were included, the value of *I^2^* was 15.8% (RR = 0.92, 95% CI = 0.87–0.98, *P* = 0.250) in fixed effects model and 15.8% (RR = 0.90, 95% CI = 0.84–0.98, *P* = 0.250) in random effects model. After excluding studies by Saposnik et al. and House et al., the value of *I^2^* was 4.9% (RR = 0.92, 95% CI = 0.86–0.99, *P* = 0.396) in fixed effects model and 4.9% (RR = 0.90, 95% CI = 0.86–0.99, *P* = 0.396) in random effects model. It means that the results were reliable and not affected markedly by these two studies (**S7 Table**). In terms of the efficacy for preventing stroke and cerebral hemorrhage in the standard meta-analysis, FA + VB6 + VB12 was more efficacious than placebo (RR = 0.86, 95% CI = 0.80–0.97; RR = 0.74, 95% CI = 0.59–0.94, respectively) (**S4 Table**). In addition, for preventing stroke, FA + VB6 + VB12 was more efficacious than FA + VB12 (RR = 0.69; 95% CI = 0.51–0.94).  **Network meta-analysis**  For the network meta-analysis, the evidence network for B vitamin supplementation for efficacy outcomes is in **Fig. 2**. The number of studies and participants was greater for placebo and FA + VB6 + VB12 groups than other combinations. The efficacy of the 8 treatments in the network meta-analysis based on the random-effects model is in **Table 1**. The risk of stroke was lower with FA + VB6 than FA + VB12 (RR_FA+VB12 vs FA+VB6_ = 0.56, 95% CrI = 0.29–1.00) and was lower with FA + VB6 + VB12 than placebo (RR_Placebo vs FA+VB6+VB12_ = 0.82, 95% CrI = 0.72–0.92). The risk of stroke was lower with FA + VB6 + VB12 than FA + VB12 (RR_FA+VB6+VB12 vs FA+VB12_ = 1.32, 95% CrI = 1.09–1.59). Furthermore, in each loop, the absolute difference between direct and indirect estimates was assessed by the IF with corresponding 95% CI. All IFs were compatible with zero (IF = 0.252, 0.237 and 0.013, respectively) and all 95% CIs included zero, which indicated that no inconsistent loops existed in each network. Heterogeneity for the indirect comparison analysis was presented by τ^2^, and a smaller value of τ^2^ indicated a better model. All τ^2^ were compatible with zero, thus heterogeneity was considered to be low. Overall, the model’s fit was relatively robust.  According to the Bayesian framework, we ranked treatments and estimated the cumulative ranking probabilities of the best treatment by the random-effects models. The treatments ranked in order of efficacy for stroke, from higher to lower, were FA + VB6 > FA > FA + VB6 + VB12 > VB6 + VB12 > niacin > VB6 > placebo > FA + VB12 (**Table 2**). The smaller number of stroke cases illustrated the probability of more effective drug treatment. A posterior mean residual deviance (total residual deviance [Totresdev] = 64.27; 95% CI = 46.39–84.98) in the random-effects model was close to the number of data points of 64 arms, which indicated very good fit of the model. | #8- #9 |

Page 1 of 2

| **Section/topic** | **#** | **Checklist item** | **Reported on page #** |
| --- | --- | --- | --- |
| Risk of bias across studies | 15 | According to Begg test, the publication bias of trials was assessed in the regular meta-analysis, including stroke group, cerebral infarction group and cerebral hemorrhage group, respectively. There was no obvious bias in each group (*P* > 0.05 was considered as no publication bias). Begg's funnel plots were shown in the Supporting Information (**S1-S3 Figs**). In addition, we performed sensitivity analysis of 22 trials for stroke prevention, in which one study was removed at a time, to evaluate reliability of the result (**S7 Table**). | #8 |
| Additional analyses | 16 | For mixed and indirect estimates, the values presented in percentile reflect the contribution of direct comparisons results to the possible pairwise comparison (**S6 Table**). | #9 |
| **RESULTS** | | |  |
| Study selection | 17 | **Fig. 1. Flow diagram of the network meta-analysis.**  17 articles (14 two-arm trials and 3 four-arm trials) were included in the network meta-analysis | #8 |
| Study characteristics | 18 | The main characteristics of the studies are in **S1 Table** [16-32]. | #7 |
| Risk of bias within studies | 19 | According to Begg test, the publication bias of trials was assessed in the regular meta-analysis, including stroke group, cerebral infarction group and cerebral hemorrhage group, respectively. There was no obvious bias in each group (*P* > 0.05 was considered as no publication bias). Begg's funnel plots were shown in the Supporting Information (**S1-S3 Figs**). In addition, we performed sensitivity analysis of 22 trials for stroke prevention, in which one study was removed at a time, to evaluate reliability of the result (**S7 Table**).  Furthermore, in each loop, the absolute difference between direct and indirect estimates was assessed by the inconsistency factor (IF) with corresponding 95% CIs (**S5 Table**). | #9 |
| Results of individual studies | 20 | Supplementation with B vitamins was associated with reduced risk of stroke and cerebral hemorrhage in the standard meta-analysis (**S3 Table**).The evidence network for B vitamin supplementation for efficacy outcomes is in **Fig. 2**. | #8 - #9 |
| Synthesis of results | 21 | Supplementation with B vitamins was associated with reduced risk of stroke and cerebral hemorrhage in the standard meta-analysis (**S3 Table**). In terms of the efficacy for preventing stroke and cerebral hemorrhage in the standard meta-analysis, FA + VB6 + VB12 was more efficacious than placebo (RR = 0.86, 95% CI = 0.80–0.97; RR = 0.74, 95% CI = 0.59–0.94, respectively) (**S4 Table**). In addition, for preventing stroke, FA + VB6 + VB12 was more efficacious than FA + VB12 (RR = 0.69; 95% CI = 0.51–0.94).  **Table 1** showed that the risk of stroke was lower with FA + VB6 than FA + VB12 (RR_FA+VB12 vs FA+VB6_ = 0.56, 95% CrI = 0.29–1.00) and was lower with FA + VB6 + VB12 than placebo (RR_Placebo vs FA+VB6+VB12_ = 0.82, 95% CrI = 0.72–0.92). The risk of stroke was lower with FA + VB6 + VB12 than FA + VB12 (RR_FA+VB6+VB12 vs FA+VB12_ = 1.32, 95% CrI = 1.09–1.59).  The treatments ranked in order of efficacy for stroke, from lower to higher, were FA + VB6 > FA > FA + VB6 + VB12 > VB6 + VB12 > niacin > VB6 > placebo > FA + VB12 (**Table 2**). | #8 - #10 |
| Risk of bias across studies | 22 | The overall study quality assessed by the Jadad score was considered high (**S2 Table**). According to Begg test, the publication bias of trials was assessed in the regular meta-analysis, including stroke group, cerebral infarction group and cerebral hemorrhage group, respectively. There was no obvious bias in each group (*P* > 0.05 was considered as no publication bias). Begg's funnel plots were shown in the Supporting Information (**S1-S3 Figs**). In addition, we performed sensitivity analysis of 22 trials for stroke prevention, in which one study was removed at a time, to evaluate reliability of the result (**S7 Table**). | #7-#8 |
| Additional analysis | 23 | The values presented in percentile reflect the contribution of direct comparisons results to the possible pairwise comparison. The range of value is 0-100%, when the value is 0, it means that the result of direct comparison does not have an effect on the other possible pairwise comparisons. When the value is 100%, it indicates that all information of the possible pairwise comparison came from the direct comparison without being influenced by other direct comparisons. The overall contribution estimates were presented by entire network, which reflected the contributions of direct evidence in the network, such as the contributions of direct comparisons 1 vs 4, 1 vs 5, 1 vs 2, 1 vs 6, 1 vs 8, 4 vs 7, 1 vs 3, 3 vs 5, 4 vs 5 and 3 vs 4 were 17.4%, 13.2%, 12.0%, 12.0%, 12.0%, 12.0%, 6.3%, 5.8%, 4.7% and 4.4%, respectively (**S6 Table**). | #9 |
| **DISCUSSION** | | |  |
| Summary of evidence | 24 | Although supplementation with B vitamins has been used for stroke prevention for a long time, the effects are still inconsistent and the comparisons of the effects of different B vitamin combinations were insufficient. We performed a standard meta-analysis to identify the impact of B vitamin supplementation on risk of stroke. Moreover, we used a network meta-analysis to estimate the optimal therapy for stroke prevention and the relationship between various combinations of B vitamin supplementation.  According to fixed- and random-effects standard meta-analysis, we found B vitamin supplementation associated with reduced risk of stroke and cerebral hemorrhage. These results were consistent with the meta-analysis by Huang et al [33], who demonstrated that B vitamin supplementation had a significant protective effect on stroke (RR = 0.88, 95% CI = 0.82, 0.95). Gao et al [34]. found that B vitamin supplementation significantly reduced homocysteine levels, which could reduce the risk of stroke events. In contrast, a recent meta-analysis based on absolute and relative measures of association showed that B vitamin supplementation could not reduce the risk of stroke [35]. Albert et al [19]. found that B vitamin supplementation might increase the risk of stroke perhaps because of different efficacy among combinations of B vitamins. However, a comparison of the efficacy of the therapies was lacking. Network meta-analysis could be a suitable method to access the interaction between various B vitamin combinations.  In our network meta-analysis of 17 randomized controlled trials involving 86 393 subjects with 3 847 stroke events, we identified the optimal therapy for stroke prevention by synthesizing outcomes in terms of the effects of B vitamin supplementation on stroke. According to the posterior probability of the Bayesian method, FA + VB6 was the top treatment among all combinations. Moreover, FA + VB6 was more efficacious than FA + VB12 and FA + VB6 + VB12 more efficacious than FA + VB12. FA + VB6 might be the optimal treatment for prevention of stroke. FA was ranked the second-best therapy; the efficacy was favorable when it was combined with VB6 but was inferior when combined with VB12. FA + VB6 + VB12 supplementation has been considered first-line treatment for stroke prevention in recent years. From our study, the efficacy may be due to the combination of FA + VB6. Thus, FA and VB6 are valuable for stroke prevention, and that of VB12 needs further investigation.  FA has an important protective role in stroke [36]. FA supplementation could reduce the risk of stroke by 18% in primary prevention [37]. The intake of FA or VB6, largely derived from multiple vitamins and fortified breakfast cereals, was highly associated with reduced levels of homocysteine [38]. The risk of stroke increased by 59% with levels of homocysteine increased by 5 μmol/L [39].  Our study suggested that FA + VB12 ranked last in efficacy. Ebbing et al. demonstrated that the risk of cardiovascular diseases could not be reduced by lowering plasma total homocysteine concentration with FA + VB12 [17]. Other studies reported that plasma concentrations of FA and VB12 were inversely related to homocysteine levels, but the efficacy of FA + VB12 on stroke prevention was not clarified [40,41]. Therefore, the efficacy of VB12 and its combinations on stroke prevention need to be further explored for clinical practice. | #10-#13 |
| Limitations | 25 | Our study contains some limitations. Despite a detailed search mechanism, we may have missed some data from unpublished studies, which might have affected our results. As well, we considered only the outcomes of interest in all eligible studies rather than the specific information for participants in different intervention groups in our network meta-analysis. | #12 |
| Conclusions | 26 | Our network meta-analysis of randomized controlled trials of the association of B vitamin supplementation and stroke indicated that FA + VB6 might be the most efficacious treatment for stroke prevention. FA and VB6 were valuable for preventing stroke, but the role of VB12 remains to be studied. The different B vitamins and their combinations had different efficacy on stroke prevention, which has significance for clinical B vitamin supplementation or human intervention. | #12 |
| **FUNDING** | | |  |
| Funding | 27 | This study was supported by the Science and Technology Plans of Guangdong Province (2013B021800264) and the Natural Science Foundation of Guangdong Province (2014A030313472). |  |

*From:*  Moher D, Liberati A, Tetzlaff J, Altman DG, The PRISMA Group (2009). Preferred Reporting Items for Systematic Reviews and Meta-Analyses: The PRISMA Statement. PLoS Med 6(6): e1000097. doi:10.1371/journal.pmed1000097

For more information, visit: **www.prisma-statement.org**.

Page 2 of 2
