# Supplementary material for: Efficacy of Supplementation with B Vitamins for Stroke Prevention: A Network Meta-Analysis of Randomized Controlled Trials
Source: PLoS One. 2015 Sep 10;10(9):e0137533. doi: 10.1371/journal.pone.0137533 (PMC4565665; doi:10.1371/journal.pone.0137533)
Supplement: S1 Table — (DOC) [file pone.0137533.s005.doc]

**S1 Table.** Main clinical characteristics of the included randomized controlled trials.

| Author | Year | Disease status | Trial design | Mean age, y | Follow-up, y | Compared Regimens | Main end point |
| --- | --- | --- | --- | --- | --- | --- | --- |
| Saposnik [16] | 2009 | CVD | Double-blind | 69 | 5 | FA + VB6 + VB12; Placebo | Stroke |
| Ebbing [17] | 2010 | Ischemic heart disease | Double-blind | 62.3 | 7 | FA + VB6 + VB12; VB6; FA + VB12; placebo | CVD, acute myocardial infarction and stroke |
| Galan [18] | 2010 | A history of ischemic heart disease or stroke | Double-blind | M 61  F 63 | 4.7 | FA + VB6; placebo | Non-fatal myocardial infarction, stroke and death from CVD |
| Albert [19] | 2008 | High-risk women with and without CVD | Double-blind | 63 | 7.3 | FA + VB6 + VB12; placebo | MI, stroke, coronary revascularization procedures and cardiovascular mortality |
| Hankey [20] | 2012 | Previous stroke or transient ischemic attack | Double-blind | 63 | 3.4 | FA + VB6 + VB12; placebo | Stroke, myocardial infarction and death from vascular causes |
| VITATOPS [21] | 2010 | Recent stroke or TIA | Double-blind | 63 | 3.4 | FA + VB6 + VB12; placebo | Non-fatal stroke, MI and death from any vascular causes |
| HPS2–THRIVE [22] | 2014 | Vascular disease | Double-blind | 50–80 | 3.9 | Niaci-Laropiprant; placebo | Stroke and death from coronary causes |
| Ebbing [23] | 2008 | Coronary artery disease or aortic valve stenosis | Double-blind | 62 | 3.2 | FA + VB6 + VB12; FA + VB12; VB6; placebo | Acute MI, all-cause death and stroke |
| Cole [24] | 2007 | History of colorectal adenomas | Double-blind | 57 | 10.25 | FA; placebo | At least 1 colorectal adenoma, advanced lesions, adenoma multiplicity and stroke |
| SEARCH [25] | 2010 | MI | Double-blind | 18–80 | 6.7 | FA +VB12; placebo | MI and stroke |
| Jamison [26] | 2007 | Advanced chronic kidney and end-stage renal disease, high Hcy levels | Double-blind | 66 | 3.2 | FA + VB6 + VB12; placebo | All-cause mortality; MI and stroke |
| House [27] | 2010 | Type 1 or 2 diabetes | Double-blind | 61 | 2.7 | FA + VB6 + VB12; placebo | MI, stroke and all-cause mortality |
| The Coronary Drug Project Research Group [28] | 1975 | Previous MI | Double-blind | 44 | 4.5 | Niacin; placebo | Coronary mortality, recurrent MI, acute coronary insufficiency, stroke and pulmonary embolism |
| Bostom [29] | 2011 | Chronic kidney disease | Double-blind | 52 | 4 | FA + VB6 + VB12; VB6 + VB12 | CVD death, MI, stroke and renal artery revascularization |
| Bønaa [30] | 2006 | Acute MI | Double-blind | 63 | 3.4 | FA + VB6 + VB12; FA + VB12; VB6; placebo | Recurrent MI, stroke and death attributed to coronary artery disease |
| Severino BImasa [31] | 2009 | Unstable angina or non–ST-elevation MI | Double-blind | 60 | 0.5 | FA + VB6 + VB12; placebo | Death and nonfatal ACS |
| Zoungas [32] | 2006 | Chronic renal failure | Double-blind | 57 | 3.6 | FA; placebo | MI, stroke and death from CVD |

ACS, acute coronary syndrome; CVD, cardiovascular disease; FA, folic acid; F, female; Hcy, homocysteine; MI, myocardial infarction; M, male; TIA, transient ischemic attack; VB, vitamin B.
