# Supplementary material for: Efficacy of Supplementation with B Vitamins for Stroke Prevention: A Network Meta-Analysis of Randomized Controlled Trials
Source: PLoS One. 2015 Sep 10;10(9):e0137533. doi: 10.1371/journal.pone.0137533 (PMC4565665; doi:10.1371/journal.pone.0137533)
Supplement: S2 Table — (DOC) [file pone.0137533.s006.doc]

**S2 Table. Assessment of study Quality (Jadad).**

| Author | Year | Randomization | Concealment of allocation | Double blind | Withdrawals and dropouts |
| --- | --- | --- | --- | --- | --- |
| Saposnik | 2009 | yes | unclear | yes | yes |
| Ebbing | 2010 | yes | unclear | yes | yes |
| Galan | 2010 | yes | yes | yes | yes |
| Albert | 2008 | yes | yes | yes | unclear |
| Hankey | 2012 | yes | yes | yes | yes |
| VITATOPS | 2010 | yes | yes | yes | yes |
| HPS2–THRIVE | 2014 | yes | unclear | yes | yes |
| Ebbing | 2008 | yes | yes | yes | yes |
| Cole | 2007 | yes | yes | yes | yes |
| SEARCH | 2010 | yes | unclear | yes | yes |
| Jamison | 2007 | yes | yes | yes | yes |
| House | 2010 | yes | yes | yes | yes |
| The Coronary Drug Project Research Group | 1975 | yes | unclear | yes | unclear |
| Bostom | 2011 | yes | unclear | yes | yes |
| Bønaa | 2006 | yes | yes | yes | yes |
| Severino BImasa | 2009 | yes | no | yes | yes |
| Zoungas | 2006 | yes | unclear | yes | yes |
