# Supplementary material for: Efficacy of Supplementation with B Vitamins for Stroke Prevention: A Network Meta-Analysis of Randomized Controlled Trials
Source: PLoS One. 2015 Sep 10;10(9):e0137533. doi: 10.1371/journal.pone.0137533 (PMC4565665; doi:10.1371/journal.pone.0137533)
Supplement: S3 Table — (DOC) [file pone.0137533.s007.doc]

**S3 Table. Fixed- and random-effects meta-analysis comparing any B vitamin supplementation** versus placebo for each outcome.

| Outcome | No. of trials | Effects model | RR (95% CI) | *I^2^* (%) | P Value for heterogeneity |
| --- | --- | --- | --- | --- | --- |
| Stroke | 22 | Fixed | 0.92 (0.87–0.98) | 15.8 | 0.250 |
|  |  | Random | 0.90 (0.84–0.98) | 15.8 | 0.250 |
| Cerebral infarction | 11 | Fixed | 0.97 (0.88–1.07) | 0 | 0.661 |
|  |  | Random | 0.97 (0.88–1.07) | 0 | 0.661 |
| Cerebral hemorrhage | 11 | Fixed | 0.74 (0.59–0.94) | 0 | 0.574 |
|  |  | Random | 0.74 (0.58–0.94) | 0 | 0.574 |

95% CI, 95% confidence interval; RR, relative risk.
