# Supplementary material for: Efficacy of Supplementation with B Vitamins for Stroke Prevention: A Network Meta-Analysis of Randomized Controlled Trials
Source: PLoS One. 2015 Sep 10;10(9):e0137533. doi: 10.1371/journal.pone.0137533 (PMC4565665; doi:10.1371/journal.pone.0137533)
Supplement: S4 Table — (DOC) [file pone.0137533.s008.doc]

**S4** Table. Efficacy in meta-analysis of direct comparisons.

| Outcome/  B vitamin treatment | Trials  n | RR | 95% CI | *P* value | *I^2^* |
| --- | --- | --- | --- | --- | --- |
| Stroke |  |  |  |  |  |
| FA+VB6+VB12 vs placebo | 11 | 0.86 | 0.80–0.97 | 0.271 | 19.6% |
| Niacin vs placebo | 2 | 0.94 | 0.85–1.05 | 0.055 | 72.9% |
| FA vs placebo | 2 | 0.79 | 0.62–1.01 | 0.142 | 53.6% |
| FA+VB6 vs placebo | 1 | 0.60 | 0.35–1.02 | – | – |
| FA+VB12 vs placebo | 4 | 1.02 | 0.88–1.17 | 0.979 | 0 |
| FA+VB6+VB12 vs VB6+VB12 | 2 | 1.11 | 0.70–1.75 | – | – |
| FA+VB6+VB12 vs FA+VB12 | 3 | 0.69 | 0.51–0.94 | 0.928 | 0 |
| FA+VB6+VB12 vs VB6 | 3 | 0.80 | 0.58–1.09 | 0.522 | 0 |
| FA+VB12 vs VB6 | 3 | 1.15 | 0.86–1.53 | 0.594 | 0 |
| VB6 vs placebo | 3 | 0.90 | 0.67–1.19 | 0.817 | 0 |
| Cerebral infarction |  |  |  |  |  |
| FA+VB6+VB12 vs placebo | 11 | 0.97 | 0.88–1.07 | 0.661 | 0 |
| Cerebral hemorrhage |  |  |  |  |  |
| FA+VB6+VB12 vs placebo | 11 | 0.74 | 0.59–0.94 | 0.570 | 0 |

95% CI, 95% confidence interval; FA, folic acid; RR, relative risk; VB, vitamin B.
