# Supplementary material for: Efficacy of Supplementation with B Vitamins for Stroke Prevention: A Network Meta-Analysis of Randomized Controlled Trials
Source: PLoS One. 2015 Sep 10;10(9):e0137533. doi: 10.1371/journal.pone.0137533 (PMC4565665; doi:10.1371/journal.pone.0137533)
Supplement: S5 Table — (DOC) [file pone.0137533.s009.doc]

**S5 Table**. Assessment of inconsistency in treatment triangle loops for stroke network.

| Closed loops | IF with 95% CI | Heterogeneity (τ^2^) |
| --- | --- | --- |
| Stroke |  |  |
| FA+VB12–FA+VB12+VB6–Placebo | 0.252 (0.00–0.61) | 0.000 |
| FA+VB12+VB6–Placebo–VB6 | 0.237 (0.00–0.68) | 0.000 |
| FA+VB12–Placebo–VB6 | 0.013 (0.00–0.68) | 0.000 |

FA, folic acid; IF, inconsistency factor; 95% CI, 95% confidence interval; VB, vitamin B.
