# Supplementary material for: Efficacy of Supplementation with B Vitamins for Stroke Prevention: A Network Meta-Analysis of Randomized Controlled Trials
Source: PLoS One. 2015 Sep 10;10(9):e0137533. doi: 10.1371/journal.pone.0137533 (PMC4565665; doi:10.1371/journal.pone.0137533)
Supplement: S6 Table — (DOC) [file pone.0137533.s010.doc]

**S6 Table. Mixed and indirect estimates for direct comparison of different treatments in the network (%)**.

|  | **Direct comparisons** | | | | | | | | | |
| --- | --- | --- | --- | --- | --- | --- | --- | --- | --- | --- |
|  | 1 vs 2 | 1 vs 3 | 1 vs 4 | 1 vs 5 | 1 vs 6 | 1 vs 8 | 3 vs 4 | 3 vs 5 | 4 vs 5 | 4 vs 7 |
| Mixed estimates |  |  |  |  |  |  |  |  |  |  |
| 1 vs 2 | 99.9 | 0.0 | 0.0 | 0.0 | 0.0 | 0.0 | 0.0 | 0.0 | 0.0 | 0.0 |
| 1 vs 3 | 0.0 | 27.3 | 14.6 | 21.2 | 0.0 | 0.0 | 13.4 | 22.3 | 1.2 | 0.0 |
| 1 vs 4 | 0.0 | 4.9 | 62.2 | 13.2 | 0.0 | 0.0 | 6.5 | 1.7 | 11.5 | 0.0 |
| 1 vs 5 | 0.0 | 6.5 | 12.1 | 60.8 | 0.0 | 0.0 | 1.9 | 8.4 | 10.3 | 0.0 |
| 1 vs 6 | 0.0 | 0.0 | 0.0 | 0.0 | 100.0 | 0.0 | 0.0 | 0.0 | 0.0 | 0.0 |
| 1 vs 8 | 0.0 | 0.0 | 0.0 | 0.0 | 0.0 | 100.0 | 0.0 | 0.0 | 0.0 | 0.0 |
| 3 vs 4 | 0.0 | 19.9 | 29.0 | 9.1 | 0.0 | 0.0 | 15.7 | 17.8 | 8.7 | 0.0 |
| 3 vs 5 | 0.0 | 21.7 | 4.8 | 26.6 | 0.0 | 0.0 | 11.7 | 28.4 | 6.8 | 0.0 |
| 4 vs 5 | 0.0 | 1.2 | 35.6 | 34.4 | 0.0 | 0.0 | 6.0 | 7.2 | 15.6 | 0.0 |
| 4 vs 7 | 0.0 | 0.0 | 0.0 | 0.0 | 0.0 | 0.0 | 0.0 | 0.0 | 0.0 | 100.0 |
| Indirect estimates |  |  |  |  |  |  |  |  |  |  |
| 1 vs 7 | 0.0 | 2.7 | 34.5 | 7.3 | 0.0 | 0.0 | 3.6 | 0.9 | 6.4 | 44.5 |
| 2 vs 3 | 38.7 | 16.8 | 8.9 | 13.0 | 0.0 | 0.0 | 8.2 | 13.7 | 0.7 | 0.0 |
| 2 vs 4 | 44.5 | 2.7 | 34.5 | 7.3 | 0.0 | 0.0 | 3.6 | 0.9 | 6.4 | 0.0 |
| 2 vs 5 | 44.3 | 3.6 | 6.8 | 33.9 | 0.0 | 0.0 | 1.1 | 4.7 | 5.7 | 0.0 |
| 2 vs 6 | 50.0 | 0.0 | 0.0 | 0.0 | 50.0 | 0.0 | 0.0 | 0.0 | 0.0 | 0.0 |
| 2 vs 7 | 30.8 | 1.9 | 23.9 | 5.1 | 0.0 | 0.0 | 2.5 | 0.6 | 4.4 | 30.8 |
| 2 vs 8 | 50.0 | 0.0 | 0.0 | 0.0 | 0.0 | 50.0 | 0.0 | 0.0 | 0.0 | 0.0 |
| 3 vs 6 | 0.0 | 16.8 | 8.9 | 13.0 | 38.7 | 0.0 | 8.2 | 13.7 | 0.7 | 0.0 |
| 3 vs 7 | 0.0 | 13.0 | 18.9 | 5.9 | 0.0 | 0.0 | 10.2 | 11.6 | 5.6 | 34.8 |
| 3 vs 8 | 0.0 | 16.8 | 9.0 | 13.0 | 0.0 | 38.7 | 8.2 | 13.7 | 0.7 | 0.0 |
| 4 vs 6 | 0.0 | 2.7 | 34.5 | 7.3 | 44.5 | 0.0 | 3.6 | 0.9 | 6.4 | 0.0 |
| 4 vs 8 | 0.0 | 2.7 | 34.5 | 7.3 | 0.0 | 44.5 | 3.6 | 0.9 | 6.4 | 0.0 |
| 5 vs 6 | 0.0 | 3.6 | 6.8 | 33.9 | 44.3 | 0.0 | 1.1 | 4.7 | 5.7 | 0.0 |
| 5 vs 7 | 0.0 | 0.8 | 22.6 | 21.9 | 0.0 | 0.0 | 3.8 | 4.6 | 9.9 | 36.4 |
| 5 vs 8 | 0.0 | 3.6 | 6.8 | 33.9 | 0.0 | 44.3 | 1.1 | 4.7 | 5.7 | 0.0 |
| 6 vs 7 | 0.0 | 1.9 | 23.9 | 5.1 | 30.8 | 0.0 | 2.5 | 0.6 | 4.4 | 30.8 |
| 6 vs 8 | 0.0 | 0.0 | 0.0 | 0.0 | 50.0 | 50.0 | 0.0 | 0.0 | 0.0 | 0.0 |
| 7 vs 8 | 0.0 | 1.9 | 23.9 | 5.1 | 0.0 | 30.8 | 2.5 | 0.6 | 4.4 | 30.8 |
| Entire network | 12.0 | 6.3 | 17.4 | 13.2 | 12.0 | 12.0 | 4.4 | 5.8 | 4.7 | 12.0 |
| Included studies | 2 | 3 | 10 | 4 | 1 | 2 | 3 | 3 | 3 | 1 |

1, placebo; 2, FA; 3, VB6; 4, FA + VB12 + VB6; 5, FA + VB12; 6, FA + VB6; 7, VB12 +VB6; 8, niacin.
