# Supplementary material for: Efficacy of Supplementation with B Vitamins for Stroke Prevention: A Network Meta-Analysis of Randomized Controlled Trials
Source: PLoS One. 2015 Sep 10;10(9):e0137533. doi: 10.1371/journal.pone.0137533 (PMC4565665; doi:10.1371/journal.pone.0137533)
Supplement: S7 Table — (DOC) [file pone.0137533.s011.doc]

**S7 Table.** **Sensitivity analysis on trials for stroke prevention.**

|  |  | Effects model | *I^2^* (%) | RR (95%CI) | *P* |
| --- | --- | --- | --- | --- | --- |
| Including studies by Saposnik et al. and House et al. |  | Fixed | 15.8 | 0.92 (0.87–0.98) | 0.250 |
|  |  | Random | 15.8 | 0.90 (0.84–0.98) | 0.250 |
| Excluding studies by Saposnik et al. and House et al. |  | Fixed | 4.9 | 0.92 (0.86–0.99) | 0.396 |
|  |  | Random | 4.9 | 0.92 (0.86–0.99) | 0.396 |

RR, relative risk; 95% CI, 95% confidence interval.
